# One-pot multicomponent polymerization towards heterocyclic polymers: a mini review

**DOI:** 10.1039/d3ra07278a

**Published:** 2024-01-08

**Authors:** Eman S. Alsolami, Hajar S. Alorfi, Khalid A. Alamry, Mahmoud A. Hussein

**Affiliations:** a Chemistry Department, Faculty of Science, King Abdulaziz University P. O. Box 80203 Jeddah 21589 Saudi Arabia mahussein74@yahoo.com maabdo@kau.edu.sa; b Chemistry Department, Faculty of Science, Assiut University Assiut 71516 Egypt

## Abstract

Multicomponent polymerization (MCP) is an innovative field related to polymer-based chemistry that offers numerous advantages derived from multicomponent reactions (MCRs). One of the key advantages of MCP is its ability to achieve high efficiency. Additionally, MCP offers other advantages, including operational simplicity, mild reaction conditions, and atom economy. MCP is a versatile technique that is used for synthesizing a wide range of analogs from several classes of heterocyclic compounds. The ring structures of heterocyclic polymers give them different mechanical, photophysical, and electrical properties to other types of polymers. Because of their unique properties, heterocyclic polymers have been widely utilized in various significant applications. MCRs are a type of chemical reaction that can be used to synthesize a wide variety of compounds in a single pot, which allows researchers to quickly assemble libraries of compounds. The development of MCPs from MCRs has made it easier to access a library of polymers with tunable structures. However, MCPs related to alkynes or acetylene triple bonds have more potential. In this review study, we provide an overview of the synthesis of heteroatom-functional polymers and alkyne-based development or other reactions such as Cu-catalyzed, catalyst-free, MCCP, MCTPs, green monomers, A^3^ coupling reactions, Passerini reactions, and sequence- and controlled-multicomponent polymerization. The up-to-date progress provides a convenient and efficient kind of approach related to heteroatoms and MCP synthesis, and perspectives in terms of future directions are also discussed in the study.

## Abstract 2

Multicomponent polymerization (MCP) is an innovative field related to polymer-based chemistry that offers numerous advantages derived from multicomponent reactions (MCRs). One of the key advantages of MCP is its ability to achieve high efficiency.

## Graphical abstract

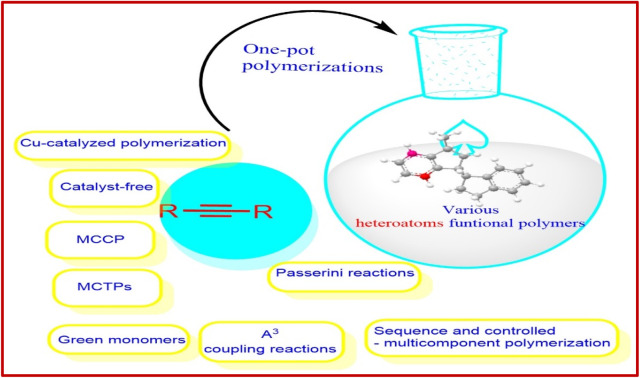

## 1. Introduction

Heterocyclic compounds are one of the largest categories related to organic-anchored substances, and they have a profound effect on human evolution. Some examples of heterocyclic compounds that are essential for human life include glucose, DNA, and vitamins B1, B2, C, and D.^1^ Long-term attempts have been made in both the fields related to academia and industry to synthesize various kind of heterocyclic polymers because of inherent benefits associated with them, including mechanical strength, specific electrical properties and processability. The incorporation of heterocycles into polymers is a rapidly evolving field. However, this field has already made a significant impact on polymer chemistry and materials science, and it is likely to play an even greater role in the future. C. S. Marvel pioneered the synthesis related to polybenzimidazoles by *in situ* producing different heterocycles in polymer backbones in the 1960s, which resulted in advancing the study related to polybenzimidazoles.^2^ Heterocyclic polymers introduce rigidity and strength into the polymer chains, which results in high glass transition temperatures. This makes heterocyclic polymers ideal for applications where high thermal stability is required, such as the aerospace industry.^1^ The advancement in the fields related to science and technology has resulted in designing and synthesis of various heterocyclic polymers with tailored properties. Heterocyclic polymers display specific thermal, mechanical, and photophysical properties as a result of the electronic effect and ring tension related to heterocyclic structures, this resulted in producing various applications in manufacturing, biological-based materials, organic photovoltaics, and light-emitting diodes, *etc.*^3^ Functional polymer science is an interdisciplinary field that encompasses polymer chemistry, materials science, biology, and the environment. It is a relatively new field, having developed in the 1960s.^4^ Modern functional polymeric materials are capable of conversion of energy, photosensitivity, chemical reactivity, and biological activity. They are crucial for modern industry and technology, with significant attention focused on developing new functional polymers to meet the demands of modern society. Heterocyclic polymers have various potential applications, but their synthesis can be challenging. Direct attachment of all the modified heterocycles to the polymer backbones that utilizes reactive heterocyclic monomers that have been synthesized is considered as a typical synthetic technique. However, the lengthy procedure related to reaction, challenging conditions in term of polymerization, challenges with numerous substituent inclusion, and affordable heterocyclic monomers usually hamper this kind of synthesis technique.^5^ However, acetylenic polymers, which are synthesized from triple-bond building blocks, possess electronically unsaturated double bonds, fused aromatic rings, or fused heterocyclic units in their main chain. Consequently, these polymers exhibit great potential as functional materials related to applications of high-technology. Polyacetylene (PA) is considered as the first acetylenic polymer that was discovered. It is also termed as a “synthetic metal” due to it good electron conductivity upon the doping factor. However, significant limitations were encountered during the initial phases of polymer science when attempting to develop polymerization related to carbon–carbon triple-bond. This was due to a number of factors, including acetylenic compounds poor availability, poor solubility and process ability of all resulting polymers. In recent decades, significant progress has been seen in the acetylenic polymerization development. This progress has been driven by advances in alkyne chemistry and materials science. In recent years, the ability to create polymers from alkyne monomers has opened up new possibilities for scientific and technological advancements. Researchers from different disciplines such as fields related to biochemistry, materials science, and engineering, *etc.* are now actively investigating the potential applications and benefits of these polymers.^6–11^ Isocyanides, as a distinctive functional group, exhibit reactivity in tetravalent and divalent carbon forms at extreme resonances. Electrophilic and nucleophilic reactions occur at divalent carbon atoms of isocyanides, leading to their conversion into tetravalent forms through exothermic reactions.^12–14^ The exceptional ability of isocyanides to construct heterocyclic compounds has attracted considerable attention in the domain of organic synthetic chemistry. The resultant products demonstrate remarkable solubility and stability, thereby presenting immense potential for application in various fields. Isocyanide-based multicomponent reactions (IMCRs) have gained recognition as powerful tools in modern chemistry, offering an efficient approach to synthesizing complex scaffolds and diverse chemical entities, including fused, bound, and spacer-linked bis-heterocyclic compounds (BHCs). In a comprehensive review by Ishwar Bhat and colleagues, important aspects such as reaction conditions, catalysts, solvents, activation sources, and the number of reported examples, along with their yield ranges, are highlighted. The showcased examples demonstrate the significant advantages of IMCRs, notably their ability to generate a wide array of BHC-containing compounds in a single reaction. Furthermore, IMCRs hold promising prospects for discovering novel BHCs with drug-like and luminescent properties, as well as their utility as building blocks for constructing metal organic frameworks and exploring other innovative applications.^15^ In addition, Wang *et al.* review recent advancements in polymerizations involving triple bonds, nitriles, and isonitriles, focusing on *in situ* reactions for heterocyclic polymer construction, offering promising prospects.^16^

Besides that, Stiernet and Debuigne review imine-based multicomponent polymerizations (MCPs) for advanced polymer design. They discuss efficiency, versatility, and the ability to create complex structures in one-pot reactions. The review highlights MCPs' maturity for applications in biomedicine, energy, and catalysis, while identifying challenges and future research directions.^17^ Also, a new kind of flexible functional films has been made by mixing biopolymers with heteroatom-doped carbon dots (CDs) for use in food monitoring and antioxidant research. The films were prepared through a convenient one-pot synthesis method, utilizing a facile physical compounding strategy in conjunction with the ‘cast and peel’ technique.^18^ MCP is a method that is used for synthesis of various polymers, including block copolymers, graft copolymers, and dendrimers. In addition to being a new research topic, MCP allows for the efficient and facile preparation related to functional polymers with complex but well-defined structures from various monomers through tandem or one-pot procedure.^19^ Many comprehensive reviews on applications and syntheses of acetylenic polymers have been published in recent years. MCRs are chemical reaction that combines three or more starting materials in a single pot to form a single product.^20,21^ MCRs are often used in organic synthesis because they can be more efficient and less time-consuming than traditional methods, have made an efficient progress in the past years, this is witnessed by the abundant reviews.^22^ For the synthesis of different compounds, most popular MCRs include reactions related to Cu-catalyzed,^23–26^ A^3^-coupling,^27^ Mannich^28–30^ and Hantzsch^31^ reactions have all been extensively researched. MCRs have a number of attractive features that make them well-suited for polymer synthesis. Polymer chemists are working hard to transform.

MCRs into MCPs with the main goal to synthesize polymers effectively with proper structures, ordered sequences related to monomer units, and potential applications.^32^ All these goals incorporate easy operation, atom economy, high efficiency rate, and little waste production.^33^ The restricted availability related to monomers, low molecular weight, and structural flaws that are brought on by various side reactions due to complexity of reactions provide significant kind of difficulties in MCPs development.^34^ Despite of all these challenges and difficulties, effective MCPs have been established, this enables the polyester,^35^ poly(ester ether ketone),^36^ poly(ester-amide),^37^ polyether,^38^ polyurethane^39^ production *etc.* green monomers are considered as natural reagent having plentiful, affordable, environmentally friendly, nontoxic, renewable, and distinctive characteristics such as oxygen (O_2_), carbon dioxide (CO_2_), and water (H_2_O), in comparison to the standard kind of monomers that are being used in MCPs. In fact, organisms can easily use MCPs to transform all the green monomers into useful kind of biopolymers present under ambient conditions. Photosynthesis is a process that occurs in plants, algae, and some bacteria, allowing them to convert CO_2_ and H_2_O in the form of glucose molecules. These glucose molecules are then polymerized to form polysaccharides. Green monomers are the one that can easily be transformed into functional polymers by the process of simple polymerizations, this draws an inspiration from the wondrous processes related to nature. For example, CO_2_ is being employed to form polyesters, polycarbonates, and polyurethanes successfully.^40–44^ However, the addition of different heteroatoms results in producing polymers having intriguing biological or photophysical properties and its use in biomedical science, filed cutting-edge optics, and smart sensing materials, *etc.*^45–47^ In this review, we present the synthesis of heteroatom polymers and alkyne or other reactions development such as Cu-catalyzed, catalyst-free, MCCP, MCTPs, green monomers, A^3^ coupling reactions, Passerini reactions, sequence-controlled multicomponent polymerization, and Ugi reactions, have gain significant attention due to their ability to offer convenient and efficient approach related to new functional materials synthesis, there is no doubt that MCP is regarded as a powerful tool that plays an important and crucial role in the functional polymers construction to meet the demands related to specialized applications. Furthermore, the advantages and disadvantages of different methods for synthesizing heteroatom-functional alkynes-based polymers are shown in Table 2 at the end of this review. It is clear that these reactions are a powerful tool that is used for functional polymers synthesis. This research offers a distinctive approach by streamlining and expediting the production of intricate heterocyclic polymers by the integration of many monomers within a single reaction step. This novel approach introduces new opportunities for the creation and production of sophisticated materials with customized characteristics and capabilities, leading to significant advancements in diverse scientific and technological fields.

## 2. The one-pot multicomponent combinatorial synthesis of heterocyclic polymers

### 2.1 Cu-catalyzed-based acetylenic synthesis of fused heterocyclic polymers

**Scheme 1:**
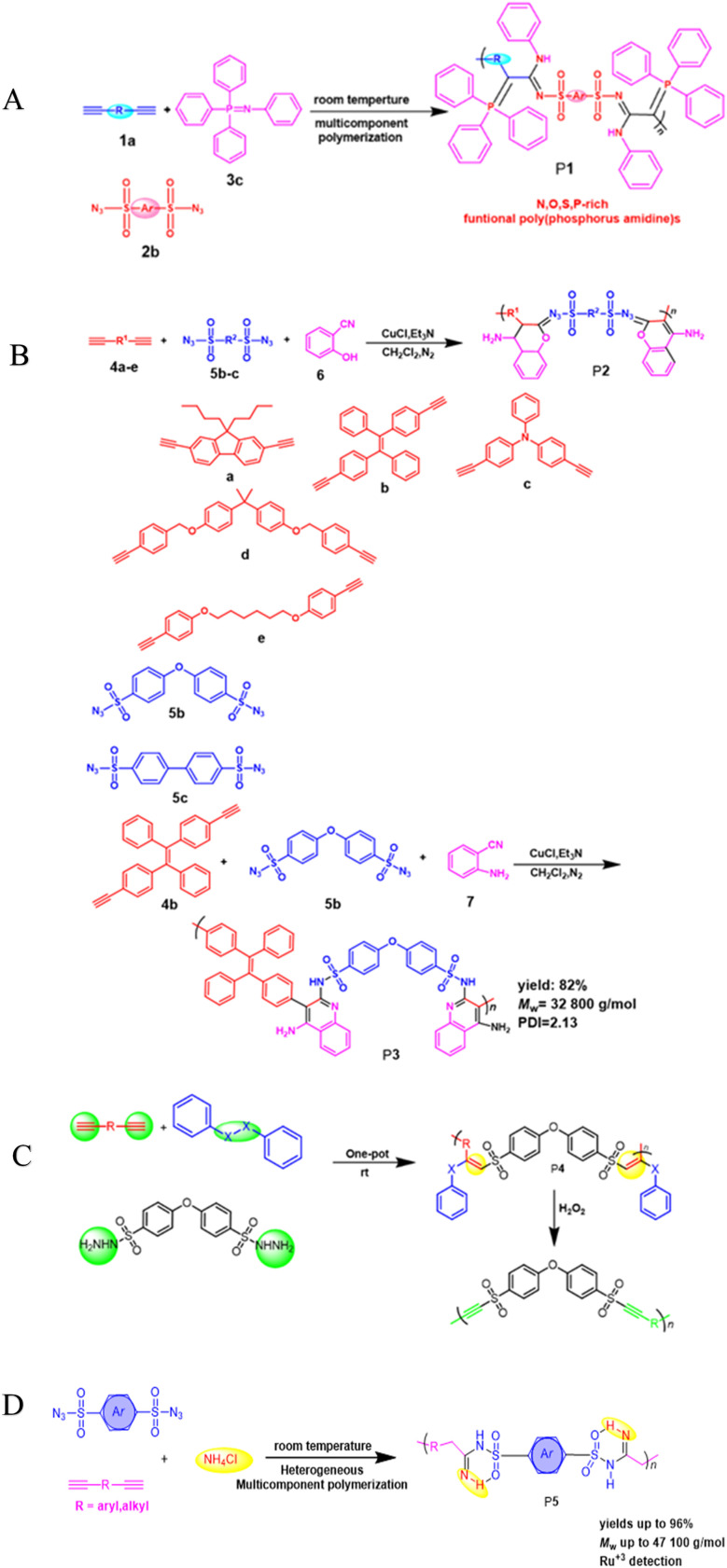
(A) Cu(i)-catalyzed polymerizations of diynes 1a, disulfonyl azides 2b, and iminophosphorane 3c generate polyphosphorus amidines P1. (B) Cu(i)-catalyzed MCPs of diynes 4a–e, disulfonyl azides 5b–c, and 2-hydroxybenzonitrile 6 or 2-aminobenzonitrile 7. (C) Cu-catalyzed MCP toward PVSs P4. (D) Cu(i)-catalyzed MCR of alkyne, sulfonyl azide and NH_4_Cl.

**Fig. 1:**
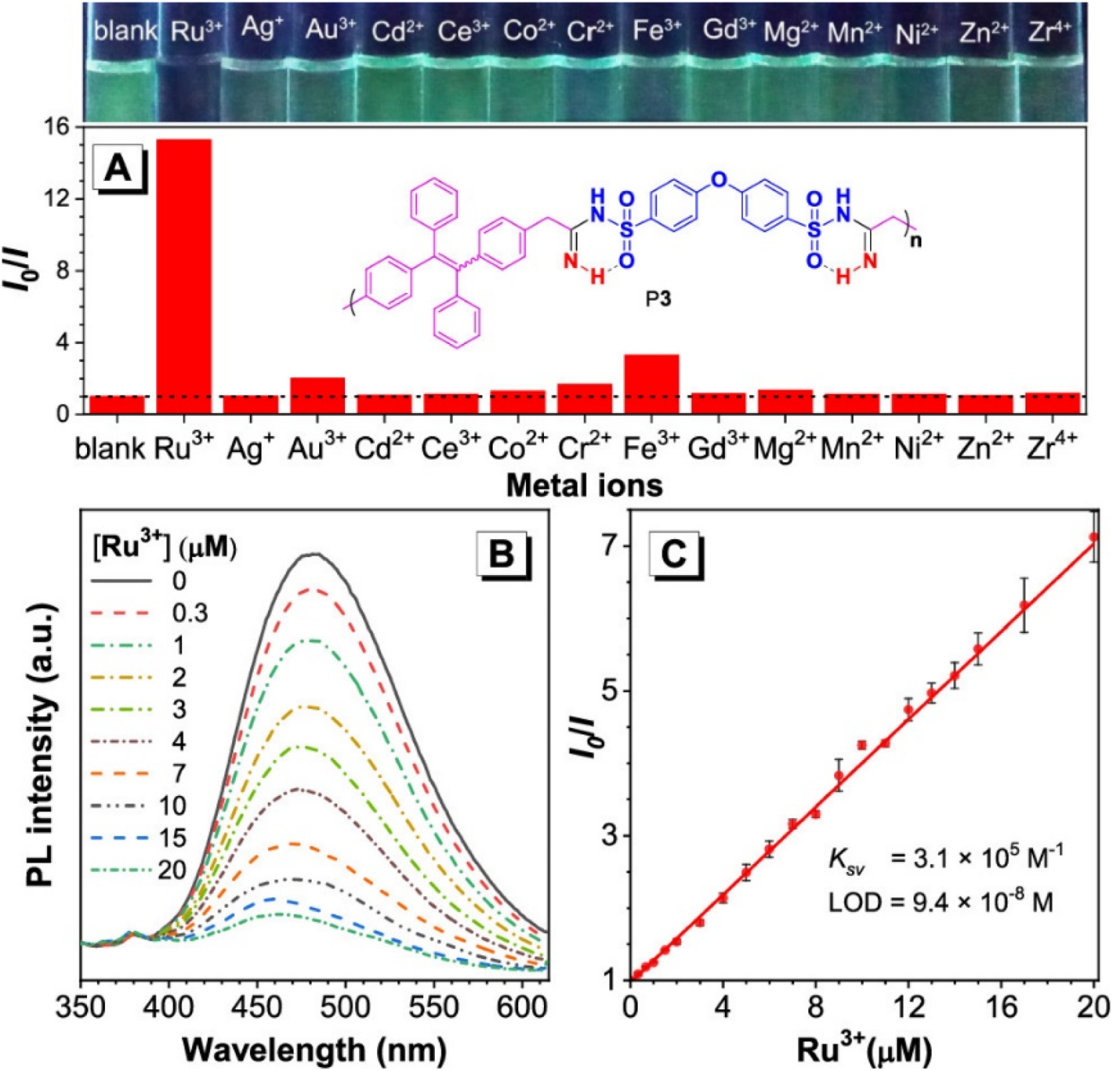
(A) Relative PL(*I*_0_/*I*) of P6 in the DMSO/water mixture with 60 vol% water contents *versus* different metal ions. *I*_0_ = PL intensity without metal ions. Polymer concentration: 6.8 mg L^−1^. Metal ion concentration: 50 μM. (B) PL spectra of P6 in the DMSO/water mixture with 60 vol% water contents in the presence of different concentrations of Ru^3+^. (C) Stern–Volmer plot of relative intensity (*I*_0_/*I*) *vs.* Ru^3+^ concentration. *I*_0_ = PL intensity without Ru^3+^. This figure has been adapted/reproduced from ref. 53 with permission from American Chemical Society, copyright 2020.

**Scheme 2:**
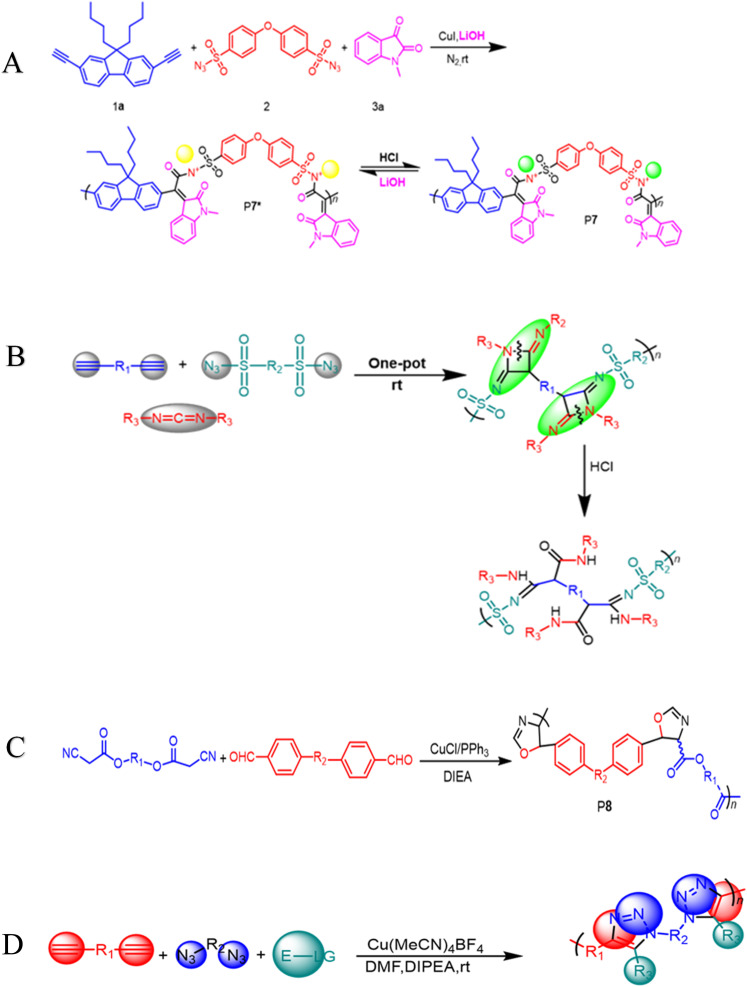
(A) The Cu(i)-catalyzed MCP of diyne 1a, disulfonyl azide 2, and N-protected isatin 3a for the synthesis of P7* and P7. (B) Cu(i)-catalyzed MCR toward 2,4-diiminoazetidine derivatives (C) dialdehydes and diisocyanoacetates were polymerized with CuI/PPh_3_/DIEA. The polymerization was carried out in DCM under nitrogen at room temperature for 6 h. (D) Multicomponent polymerization towards 1,4,5-polytriazoles.

**Scheme 3:**
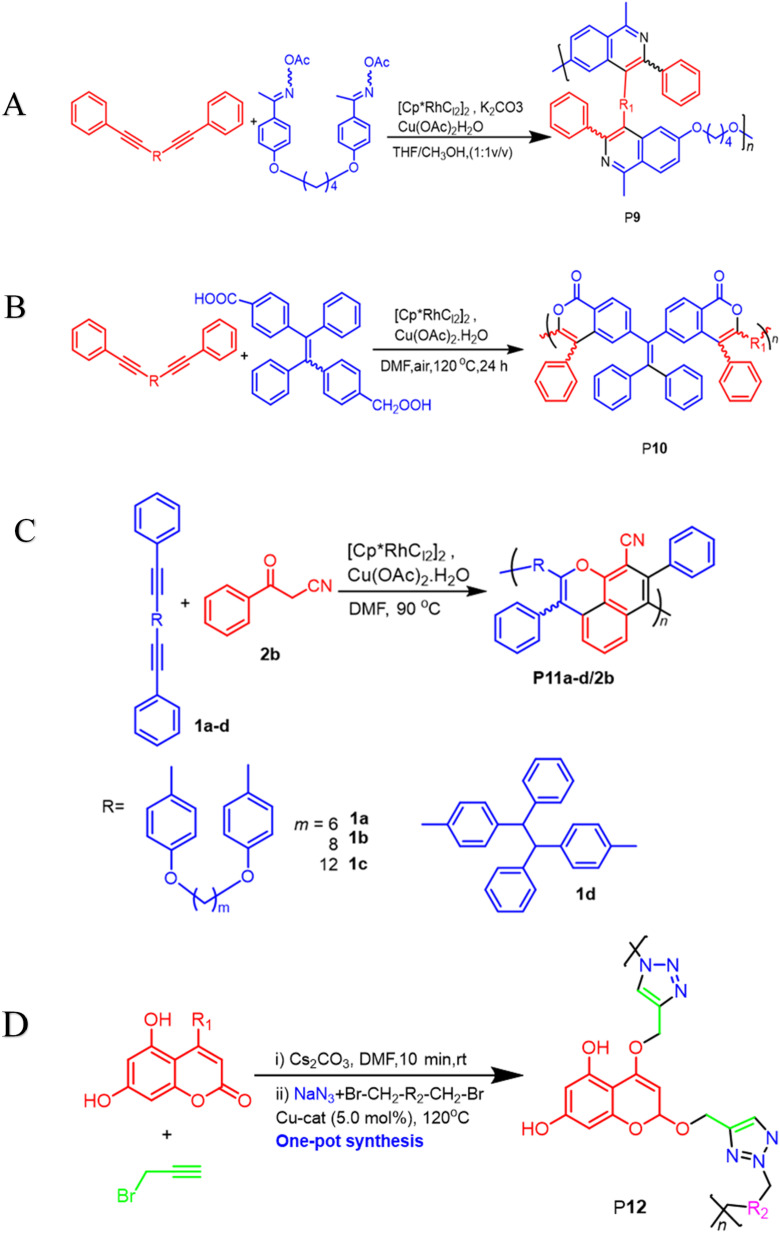
(A) A transition-metal-catalyzed two-component polycoupling of internal diynes and an *O*-acyloxime derivative towards poly(isoquinoline)s P9 (B) rhodium and copper-catalyzed oxidative polycoupling of aryl diacid and internal diyne-generating isocoumarin-containing polymers P10 (C) a cascade oxidative polyannulation of diynes and benzoylacetonitrile towards multisubstituted poly(naphthopyran)s P11a–d/2b (D) a two-component click polymerization one-pot synthesis using *O*-alkylation, sodium azide, and alkyl/benzyl dibromides produces coumarins containing 1,4-polytriazols P12.

Heterocyclic polymers are a class of polymers that contain one or more than one heteroatom, for instance, oxygen, nitrogen or sulfur, in their backbone.^48^ These heteroatoms can impart a variety of properties to polymers, including electrical, mechanical, and photophysical properties. The unique properties of heterocyclic polymers make them ideal for various applications, but the synthesis of heterocyclic polymers is an intricate process. Heterocyclic polymers are commonly synthesized using traditional methods that entail the direct attachment of heterocycles to the backbone of polymers. This can be a complicated process, and it is often limited by the following factors: the limited number of heterocyclic monomers and their low cost, complex multi-step processes, difficulties in multiple substituent incorporation, *etc.* The triple-bond-reliant polymerization process involves polymerizing monomers with triple bonds through a polymer backbone, which can then be utilized to form a heterocycle within the polymer backbone. This strategy offers advantages over traditional methods for synthesizing heterocyclic polymers. Aliphatic and aromatic heterocycles are two categories related to heterocyclic compounds. The most fundamental aliphatic heterocycle is piperidine, with six members, while its aromatic form is pyridine. Derivatives of these heterocycles often change carbon atoms or nitrogen to heteroatoms like nitrogen, sulfur, and oxygen. Six-membered heterocycles have higher stability rates and resistance to ring-opening processes.^49^ Chemists widely prefer the one-pot synthesis strategy for its ability to improve reaction efficiency and yield. This is because all starting materials react in a single reactor, eliminating the need for time-consuming purification of intermediates and tedious synthetic procedures. The method is particularly beneficial for multicomponent one-pot polymerizations, simplifying the process. For example, the copper-catalyzed one-pot three-component polymerization (Scheme 1A).^50^ This method efficiently prepares polymers that contain heteroatom N, O, S, and P with multiple functionalities. Diynes 1a, disulfonyl azides 2b, and iminophosphorane 3c polymerize smoothly under mild conditions at room temperature, producing regular and high molecular weight poly(phosphorus amidine)s P1. These polymers have a maximum weight of 85 600 g mol^−1^ and a *M*_w_/*M*_n_ ratio of 2.89, with an excellent yield of up to 92%. The model compounds of phosphorus amidine showcase both properties of thermally activated delayed fluorescence, which is normally activated through heat, and aggregation-induced emission (AIE). These distinct features offer exceptional functionalities, which include high refractive indexes, fluorescence, and the ability to detect Pd^2+^ ions sensitively.^50^ One of the monomers can be subjected to modification, which, results in a straightforward yet a systematic and efficient technique to creating functional polymers containing heterocycles or atoms of S, N, or O was developed.^51^ These polymers were synthesized in a one-pot reaction involving either of 2-hydroxybenzonitrile 6 or 2-aminobenzonitrile 7, together with diynes 4a–e, disulfonyl azides 5b–c, and were catalyzed by copper chloride and trimethylamine at room temperature (Scheme 1B).^51^ The resulting poly(*N*-sulfonylimine)s P2 had high molecular weights (up to 37 700 g mol^−1^) and a polydispersity index (PDI) of 2.85, and released only N_2_ as a byproduct, meeting the principles of green chemistry. The yields were also noteworthy, with successful harvests producing up to 96%. In the MCP reaction, the polymerization of 4b, 5b, and 7 was proven to be successful with a yield of 82% within 0.5 hours. This was achieved by substituting 2-hydroxybenzonitrile 6 with 2-aminobenzonitrile 7. The outcome resulted in the formation of poly(*N*-sulfonylimine)s P3 that has aminoquinoline, a molecular weight of 32 800 g mol^−1^, and a PDI of 2.13. The benefits of this polymerization method are numerous, including its high atom detection efficiency, its resistance to temperature changes, its unique fluorescence, its selectivity in recognizing Ru^3+^, and its antibacterial qualities. Synthetic polymerization methods that incorporate multiple chalcogen chemical components are scarce due to ineffective synthetic methods. In the realm of polymer chemistry, a noteworthy leap was taken last year. A novel and inventive approach to MCP was introduced, which resulted in the triumphant integration of four separate chalcogens into polymers.^52^ The atoms of chalcogen, especially sulfur, are responsible for the high index of refraction towards poly(vinyl sulfones) (PVSs) P4. The values of PVSs are capable of being altered from 1.8898 to 1.5845 in a span of 400–900 nm by combining different monomers. These polymers have a propensity to imbue and release into the solid state, which could be employed to create fluorescent films that are uniform and intended for use in displays, optical memories, and imaging. They have a capacity for redox reactions and can be augmented through processes of oxidization. The procedure of treating hydrogen peroxide to the selenium in PVSs is effective at removing the metals (Scheme 1c).^52^ Besides that, Huang *et al.*, (2020), developed a new synthetic technique for the heterogenous O, N, and S advanced functional polymers construction through using inorganic salts NH_4_Cl *via* one-pot click polymerizations of diyne, disulfonyl azide, and NH_4_Cl.^53^ The polymerization process were further catalyzed by CuI and Et_3_N and carried out in CH_2_Cl_2_/tetrahydrofuran at room temperature under a nitrogen atmosphere in the presence of a basic medium, affording poly(sulfonyl amidine)s P5 with high molecular weights (up to 47 100 g mol^−1^, *M*_w_/*M*_n_ = 3.08) in excellent yield (up to 96%) (Scheme 1D). Due to the ruthenium amidine moiety coordination action, the P6 usually exhibits impressive fluorescence quenching responses to Ru^3+^ and mostly functions as an effective fluorescent-based chemosensor. Under the same conditions, this polymer has no fluorescent reaction to other metal ions such as Ag^+^, Au^3+^, Cd^2+^, Ce^3+^, Fe^3+^, Gd^3+^, Mg^2+^, Mn^2+^, Ni^2+^, Zn^2+^, and Zr^4+^, this indicates a high selectivity level toward Ru^3+^ as shown in Fig. 1A–C. As ruthenium compounds are used as catalysts for material creation, the selective detection of Ru^3+^ is considered crucial. But more exposure to Ru^3+^ and direct interaction with individuals can result in serious harm to their health.^53^ In addition to the previously mentioned click related to polymerizations in alkyne-based MCP, through palladium-catalyzed polycouplings of aryl boronic acids and internal diyne, perfluoroalkyl diodes in THF/H_2_O mixtures at 39 °C for 12 h under nitrogen atmosphere, this can effectively produce a series of multi-substituted fluoropolydienes affording high molecular weight (*M*_w_ up to 69 400, *M*_w_/*M*_n_ = 4.2), were formed in high yields (up to 90.3%), furnishing the predominant *E*-configuration. By using the technique related to spin-coating, polymers can be transformed easily into a thin luminous film, this can be done easily due to AIE feature from the Tetra Phenyl Ethane (TPE) moiety that is inserted in the structures. By using a copper photomask and UV-based light to irradiate the thin layer, a high-resolution 2D fluorescent photopattern was produced successfully.^54^ For instance, Xu and co-workers developed a powerful MCP that provides straightforward access to fabricate heteroatom such as S, O, N and P-rich HBPs *in situ* using multifunctional alkynes 1a, disulfonyl azides 2, and commercially available N-protected isatins 3a act as nucleophiles. The process proceeds smoothly at room temperature or 30 °C with inexpensive catalysis of CuI, LiOH, or Na_2_CO_3_. After polymerization, HCl was easily be used to protonate the ionized polymer P7*, the resultant poly(*N*-acylsulfonamide)s P7 show high weight-average molecular weight (*M*_w_ up to 30, 600 g mol^−1^, *M*_w_/*M*_n_ = 1.98) and well defined structures(Scheme 2A). The polymers demonstrated excellent solubility, excellent film-forming ability, and increased refractivity. When LiOH and HCl were used, they underwent structural-based alterations that gave them reversibly tunable hydrophilicity as well. Notably, when the MCP is being carried out in the process of DMF along with CuI acting as the catalyst and Na_2_CO_3_, this act as the base, water is also added as the fourth component. Some poly(*N*-acylsulfonamide)s that contain oxindoles results in yellow to red emission when solid, and they have acceptable thermal stability with *T*_d_ values at 319 °C in 5 wt% weight loss under nitrogen gas.^55^ Han *et al.* (2018) developed a facile MCP method that uses terminal alkynes, disulfonyl azides, and Schiff bases as monomers. They demonstrated that the MCP method can be used to synthesize polymers with multi-substituted azetidine rings in the backbone. The MCP method that they developed uses terminal alkynes, disulfonyl azides, and Schiff bases as monomers. The monomers are first coupled to form azetidine rings, which are then polymerized using CuI as a catalyst. The polymers that are produced have high molecular weights, and they yield around 88.5% (Scheme 2B). Through an effective reaction related to acid-mediated ring-opening, the azetidine structure's distinctive ring tension enables the polymers to be easily converted into amidine or amide derivatives. This reaction is highly efficient, reaching 100% conversion within 30 minutes. The reaction usually benefits from strong functional group tolerance and good atomic economy. The azetidine structure's distinctive ring tension helps the polymers to be converted into amide and amidine derivative polymers efficiently, this reaches around 100% conversion in around 30 minutes. This characteristic usually broadens the architectures of various polymers, this allows for the construction of functional structures with adjustable properties. The research introduces a novel method for generating azetidine polymers, which is atom-efficient and exhibits strong functional group tolerance, making them easy to build, modify, and functionalize *in situ*.^56^ The electron-withdrawing effect related to the carbonyl group activates the isonitrile group in the monomer; as a result, the reaction can easily be achieved and carried out within 2 hours at room temperature. The resulting polyimidazole polymers had high molecular weights (up to 32 500 g mol^−1^), an increased yield rate (around 94%), with excellent solubility. This polymerization related to single-component has the benefit of being a straightforward synthetic process free from all the influences related to monomer-based ratios.^57^ Cheng and colleagues created another isocyanides polymerization process, the one starting from diisocyanoacetate by combining the compound with dialdehydes in CuCl/triphenylphosphine (PPh_3_)/*N*,*N* diisopropylethylenediamine (DIEA) in dichloromethane (DCM) presence. At room temperature, the reaction can easily be completed in 6 hours with an excellent yield (up to 97%) and moderate molecular weights of P8 that contains the oxazoline compounds (Scheme 2C).^58^ Zheng *et al.* suggest that 1,4,5-polytriazoles can be synthesized using multicomponent polymerization and interrupted click synthesis, employing diynes, diazides, and electrophiles. This method yields high yields, *M*_n_ values, and excellent modification efficiency when utilizing Cu(MeCN)_4_BF_4_ catalyst, in base conditions and DMF serves as a solvent (Scheme 2D). It should be noted that inorganic CuI is not a suitable catalyst for this polymerization due to its poor solubility.^59^ A transition-metal-catalyzed two-component polycouplings method has been developed for the synthesis of functional poly(isoquinoline)s. The method involves polyannulations of internal diynes and *O*-acyloxime derivative in methanol/THF mixture at 105 °C, with [Cp*RhCl_2_]_2_ and Cu(OAc)_2_H_2_O as catalysts and oxidants, producing poly(isoquinoline)s P9 in almost quantitative yields (Scheme 3A). This polymerization is suitable for various functional groups and can be carried out in a monomer nonstoichiometric balance circumstance. Highly emissive poly(isoquinoline)s with tetraphenylethene units generates photopatterns and functions as a sensitive fluorescent chemosensor for nitro-aromatic explosives.^60^ An atom-economical polymerization method using internal diyne and aryl diacid can produce functional isocoumarin-containing polymers P10. This process can be performed under nitrogen or air without loss of efficiency. In the presence of [Cp*RhCl_2_]_2_ and Cu(OAc)_2_H_2_O in dimethylformamide, a polymer with high molecular weight(*M*_w_ up to 42 900, *M*_w_/*M*_n_ = 3.99) was generated in high isolated yield (Scheme 3B). The polymer exhibits exceptional thermal stability, optical transparency, and film-forming capabilities, with high and UV-tunable refractive indices. It can create a two-dimensional fluorescent photo-pattern by irradiating a thin film with UV light, resulting in intense emission.^61^ A cascade oxidative polyannulation approach utilizing convenient diynes and commercially accessible benzoylacetonitrile was developed to generate multi-substituted poly(naphthopyran)s (PNPs) P11a–d/2b. The procedure was catalyzed in DMF at 90 °C by [Cp*RhCl_2_]_2_ and Cu(OAc)_2_H_2_O, resulting in polymers with high molecular weights and atom economy (Scheme 3C). PNP containing tetraphenylethene was emissive in solid state and increased in response to shear force and vapor fuming. PNPs are potential for application in fluorescence detectors for mechanical flaws and volatile organic substances, as well as security materials, due to their distinctive features.^62^ In order to synthesize heterocycle-based polymers, novel coumarins containing 1,4-polytriazols P12 were synthesized by a two-component click polymerization method. During the process, *O*-alkylation, sodium azide, and alkyl/benzyl dibromides were use (Scheme 3D). Several high-molecular-weight polymers with good yields were synthesized, resulting in effective dye adsorbents with high selectivity.^63^ He Junnan, *et al.* developed Cu(i)-catalyzed MCP using diynes, sulfonyl azides, and a series of tumor microenvironment (TMEs)-responsive, cleavable diols to produce biodegradable poly(*N*-sulfonylimidate)s that respond differently to tumor microenvironments, exhibiting excellent degradation performance and high molecular weights. The conjugation of tetraphenyl porphyrin to polymer side chains enhances fluorescence intensity and singlet oxygen quantum yield for photodynamic therapy.^64^ Additionally, a new synthetic methodology for imidazole-based cross-conjugated polymers has been developed using bimetallic Cu(i)/Rh(ii) relay-catalyzed multicomponent polymerization. This process uses readily available monomers like aryl alkynes, azides, and aryl nitriles. The process starts with Cu-catalyzed azide–alkyne cycloaddition (CuAAC), followed by rhodium-catalyzed ring-opening/transannulation of triazole intermediates. The new polymers have high molar mass, broad band gaps, and tunable optical properties, making them ideal for organic electronic materials.^65^

Wang *et al.* created facile access to diverse functional polymers with multisubstituted small heterocycles for MCP using difunctionalizable alkynes, sulfonyl azides, and Schiff bases. The polymers demonstrated high stereoselectivity and stability, even after treatment with HCl and KOH. The azetidine-derivative heterocycles can serve as chemosensors for Pd^2+^ and Cr_2_O_7_^2−^ detection.^66^

### 2.2 Catalyst-free based acetylenic synthesis of fused heterocyclic polymers

**Scheme 4:**
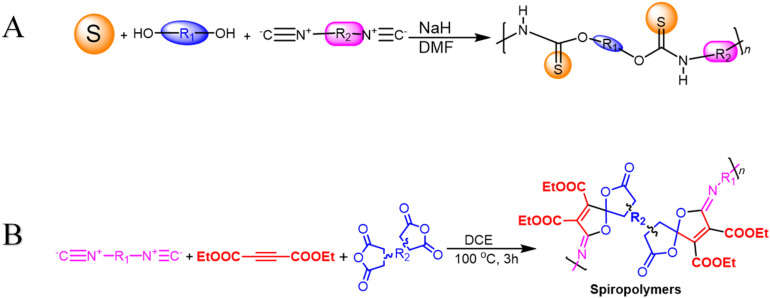
(A) Multicomponent polymerizations of sulfur, diols, and diisocyanides (B) multicomponent spiropolymerization of diisocyanides, activated alkynes, and bis-anhydrides.

**Fig. 2:**
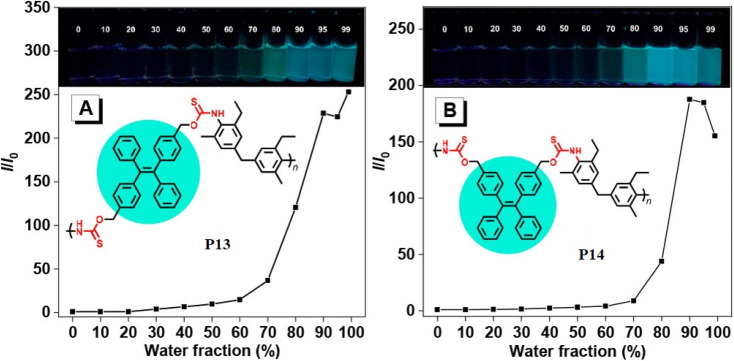
Plots of relative PL intensity *versus* water fractions (by volume) in THF/water mixtures of (A) P13 and (B) P14. Concentration of polymers: 10 μM. Excitation wavelength: 360 nm. This figure has been adapted/reproduced from ref. 69 with permission from American Chemical Society, copyright 2021.

**Fig. 3:**
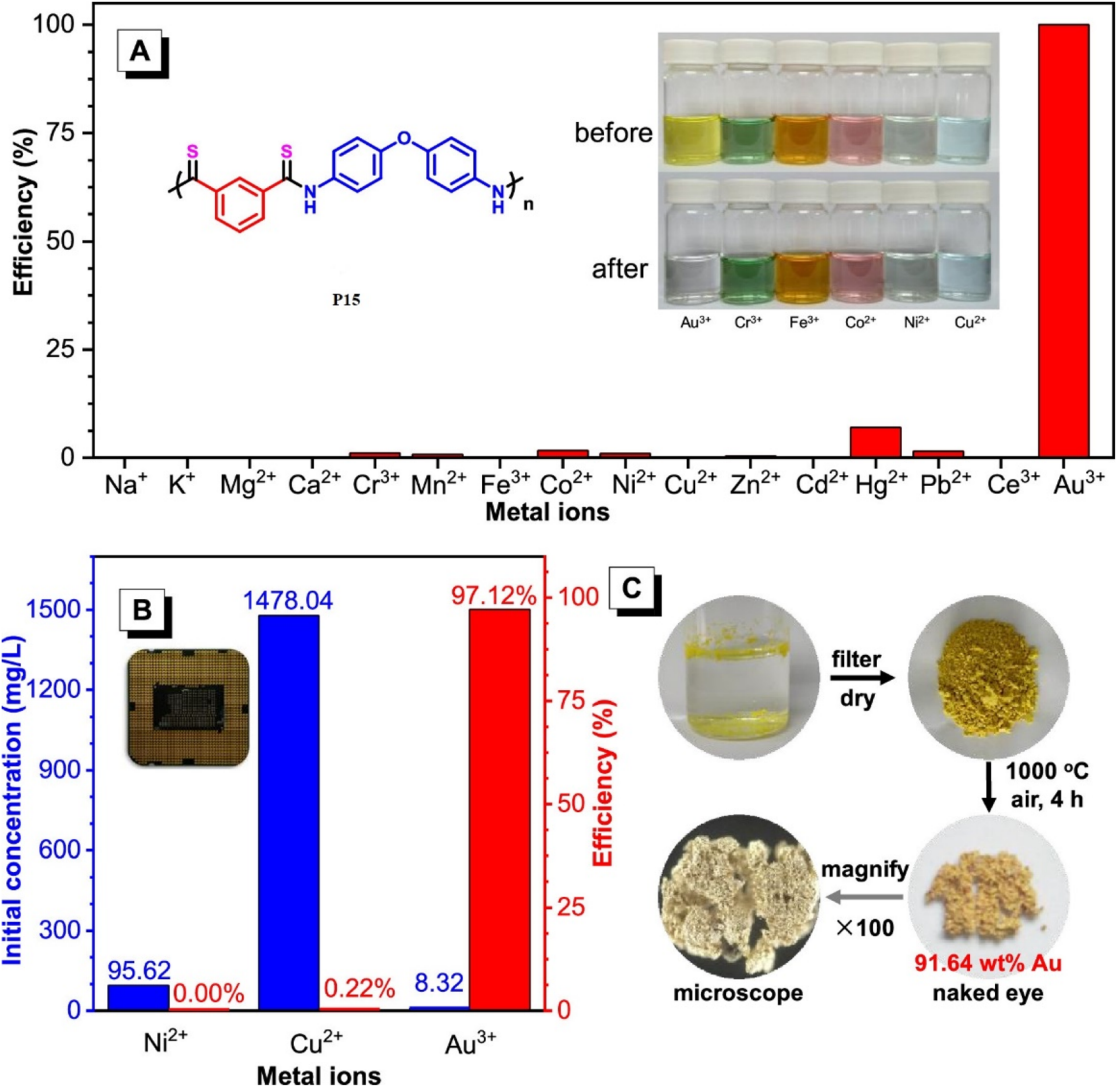
(A) Selective extraction of Au^3+^ by P15 in an aqueous solution with 16 mixed metal ions. Inset: six colored metal ion solutions before and after the treatment with P11. [Mn^+^]^0^ = 100 mg L^−1^, VMn^+^ = 10 mL, mP11 = 10 mg. (B) The initial concentrations of Ni^2+^, Cu^2+^, and Au^3+^ and the selective extraction of Au^3+^ by P15 from the leaching solution of a discarded CPU. (C) The gold recovery process with P15. This figure has been adapted/reproduced from ref. 70 with permission from American Chemical Society, copyright 2020.

Transition-metal-free polymerization is a challenging field that necessitates the development of innovative mechanisms that don't rely on transition metals. It offers several advantages, including being environmentally friendly, being conducted under milder conditions, saving energy, reducing accident risks, and controlling properties. However, it requires the development of new and innovative polymerization mechanisms.^67^ Metal-free click polymerization and reversible addition–fragmentation chain-transfer (RAFT) polymerization are common transition methods used to create various polymers due to their cost-effectiveness, safety, and desirable properties, as metal catalysts are expensive, toxic, and their optical and electrical properties are typically affected by metal catalyst residues.^68^ Seven poly(*O*-thiocarbamate)s was obtained through this MCP. After optimizing the temperature, solvent, monomer feeding ratio, and concentration of the MCP related to sulfur, diols, diisocyanides, and sodium hydride as a catalyst (Scheme 4A), the outcomes in terms of optimum polymerization were obtained successfully. The MCP reaction was conducted in *N*,*N*′-dimethyl formamide (DMF) under nitrogen at 55 °C for 1 hour, with a 4 : 4 : 1 monomer ratio of sulfur, sodium hydride, and diol. The reaction produced a soluble polymer with a large molecular weight (*M*_w_) of up to 53 100 g mol^−1^ in 95% yield. These polymers possess high refractive indices (RIs) above 1.7 have been shown to be promising materials for fluorescent sensing of harmful metal cations. The addition of TPE moieties to polymers enhances their sensitivity and selectivity (P13 and P14) in detecting metal cations like Hg^2+^, as demonstrated in Fig. 2A and B.^69^ The structure of MCR, a polymer with similar elemental sulfur in monomers, depends on the reaction conditions. In 2019, Cao *et al.* reported a catalyst-free MCP of elemental sulfur, dicarboxylic acids, and diamines. This MCP is a one-pot reaction that constructs 12 polythioamides with great structural diversity, large molecular weights (*M*_w_ up to 86 200 g mol^−1^), and excellent yields. The monomers used in this MCP are all commercially available or have abundant natural sources, making it a scalable and economically viable process. The reaction is also relatively mild, taking place at 60 °C in pyridine. Furthermore, the extraction efficiency of Au^3+^ (>99.99%) and Hg^2+^ (86.01%) of P15 and Au^3+^/Hg^2+^ was observed. The results indicate that P15 was able to selectively extract Au^3+^ from a mixed solution of 16 different metal ions with an extraction efficiency greater than 99.99%, whereas the extraction efficiency for Hg^2+^ is only 7.01% and for the other metal ions it is below 1.71%. Regarding the colored solutions, the (Fig. 3A–C) below show that Cr^3+^, Fe^3+^, Co^2+^, Ni^2+^, and Cu^2+^ showed no significant change, although the color faded for Au^3+^ exclusively. Polythioamides can, therefore, recover Au^3+^ under practical acidic conditions rapidly, efficiently (>99.99%), selectively, and with increased capacity (0.60 g Au^3+^ per g). Sulfur-containing polymers have a number of potential advantages over traditional polymers, including high performance, economy, and functionality, which have attracted significant interest in recent years.^70^ Aromatic polythioamides with intriguing features and a unique ability to coordinate metals are a type of sulfur-containing polymer that has attracted significant interest in recent years. Polythioamides can, therefore, recover Au^3+^ under practical acidic conditions rapidly, efficiently (>99.99%), selectively, and with increased capacity (0.60 g Au^3+^ per g). Yang Hu *et al.* (2023) reported the functional aromatic polythioamides synthesis from a wide and economical source of elemental sulfur, aromatic diamines, and aromatic dialdehydes in the presence of potassium hydroxide and pyridine at 110 °C with high molecular weights of up to 54 700 g mol^−1^ and high yields. The MCP method was utilized to synthesize polythioamides, which are highly effective for gold extractions due to their excellent thermal stability, solubility, and high thin film-light refractivity.^71^ As well, in a similar manner, MCP of benzoxazine–isocyanide and thioacetamide as a suitable sulfur source at room temperature (RT) generates phenolic linear polythioamides (PTAs). Further, during post-polymerization, polybenzoxazine was also modified to yield a thioamide segmented polymer. Cross-linked polythioesteramide derivatives were established by mixing the representative that produced polythioesteramides with trimesoyl chloride (TMC). Due to various affinitive units of Pb^2+^ (thioamide, amide, phenolic hydroxyl, tertiary amine, and carboxyl) in a cross-linked polythioesteramide structure, this material was also investigated as a Pb^2+^ electrochemical probe. Through this work, novel PTAs can be produced in mild conditions, modified structurally, and functionally applied in innovative methods.^72^ There has been a recent interest in the selenium-containing polymers development due to their potential applications in a variety of fields. Wu *et al.*, (2021) synthesize alicyclic poly(oxaselenolane)s from elemental selenium, diisocyanides, and dipropargyl alcohols showing good yields, thermal stability, and processability at room temperature in THF serve as solvent.^73^ Peng *et al.*'s (2022) study demonstrated that reaction customization can be utilized to explore one-pot Se-based polymerizations, and selenium (Se) is converted directly into functional polymers with unique heterocycles containing Se. The organic reactions of Se and alkynone were modified, and the reaction conditions, including the base, solvent, temperature, and procedure, were tailored for the synthesis of polymers to use elemental Se as the cheap source for the *in situ* construction of Se-containing heterocycles in the polymer backbone. In DMF that involves using Cs_2_CO_3_ as the base, effective transition metal-free room-temperature polymerization of Se and alkynones was quickly established; this yielded well-defined regioselective poly(1,4-diselenin)s with high molecular weights. To efficiently develop aromatic polyselenophene, one-pot cyclization–oxidation and two-step tandem polymerization related to selenium and alkynone were also accomplished. Such polymerizations exhibited high selectivity, increased efficiency levels, and environmental benefits. They also created nonaromatic 1,4-diselenide or aromatic selenophene heterocycles *in situ* in the polymer main chain under specific circumstances. It also resulted in creating multiple new bonds as well as a heterocycle in each polymer repeating unit. There were two distinct alkyne moieties and Se atoms in each reaction. Poly(1,4-diselenide)s were straightforwardly changed to polyselenophenes after oxidation, and the different outflow qualities of these polymers enabled constant fluorescence observation at room temperature. Moreover, poly(1,4-diselenin)s and polyselenophenes showed exceptional dissolvability, processability, chemical stability, a unique property in terms of photophysical properties, and a high refractive index (up to 1.8487 at 633 nm). This is possible due to their distinctive structural features, which make them promising useful materials.^74^ Recently, Zhu *et al.* (2022) presented a comprehensive investigation on the synthesis of the spiropolymer, incorporating the compound benzophenone. This synthesis was achieved through a metal-free multicomponent spiropolymerization (MCSP) process conducted at room temperature. The reaction involved the combination of diisocyanides, activated alkynes, and bis-anhydrides under conditions that were not excessively harsh (Scheme 4B). It is noteworthy to mention that spiropolymers were obtained with significant yields (up to 92%), exhibiting elevated molecular weights (*M*_w_ up to 92 600 g mol^−1^), and demonstrating notable thermal stability. The compound demonstrates favorable solubility along with aggregation-induced emission properties, which contribute to its notable performance in degrading under UV irradiation. Consequently, it holds promise as a viable component for photoresist materials. These polymers possess an iminofuran ring that is susceptible to degradation when exposed to strong acids. Conversely, some polymers exhibit enhanced degradation efficacy owing to their water solubility. Based on the findings, it can be concluded that they exhibit low cytotoxicity and possess the potential to serve as drug carriers for safeguarding the gastric mucosa against acid-induced release. Hence, this chemical reaction facilitates the expansion of MCSP reactions capable of synthesizing multifunctional spiropolymers, thereby expediting the advancement of polymerization techniques.^75^ Moreover, Ren *et al.* (2022) conducted a study wherein they successfully synthesized a range of poly(1,4-dihydropyridine)s (PDHPs). This was achieved through a metal-free multicomponent polymerization process involving diacetylenic esters, benzaldehyde, and aniline derivatives, all combined in a one-pot reaction. The PDHPs lacking conventional luminescent units were imbued with adjustable triplet energy levels through the phenomenon of through-space conjugation resulting from the formation of various cluster sizes. The presence of large and compact clusters has been observed to significantly enhance the extension of the phosphorescence wavelength. Room-temperature phosphorescence can be achieved by employing benzophenone as a rigid matrix to stabilize the triplet excitons. The nonconjugated polymeric clusters have the ability to exhibit phosphorescent emission with a wavelength of up to 645 nm. An experimental investigation using a combination of static and dynamic laser light scattering techniques to gain a deeper understanding of the structural characteristics of clusters formed within the host matrix melt.^76^ In addition to this, Dong *et al.* (2021) performed a study in which they observed a catalyst-free and spontaneous Michael addition polymerization process for nonconjugated poly(β-aminoacrylate)s. This process involved the utilization of terminal diyne, diisocyanates, and imidazoles as monomers and was carried out at room temperature. The poly(β-aminoacrylate) obtained without conventional AIE agents exhibited characteristics, displayed the characteristic behavior of AIE, and demonstrated notable fluorescence properties that were dependent on the excitation wavelength. These observations can be attributed to the emissions triggered by clusterization. The observed increase in the fluorescence quantum yield (Φ*F*) was directly proportional to the polymer concentration in tetrahydrofuran (THF) solutions. Specifically, the Φ*F* values increased from 5.54% at a concentration of 10 × 10^−6^ m to 10.13% at a concentration of 1 × 10^−3^ m.^77^ Furthermore, a catalyst-free, one-pot multicomponent spiropolymerization technique was developed for synthesizing spiropolymers using diisocyanides and alkynes, with CO_2_ as the monomer. The resulting spiropolymers have high weight-average molecular weights and a maximum yield of 87.7%, with a spiro structural unit in the main chain, specifically 1,6-dioxospiro^4^ nonane-3,8-diene, accompanied by various types of spacer groups. The newly developed MCS reaction can be considered an atom-economic polymerization process. It holds promise as an initial step in the synthesis of spiropolymers and exhibits significant potential for various applications in the field of materials science. This mainly reflects that a catalyst-free method has been developed for spiropolymer synthesis, yielding a higher-molecular-weight with potential applications within the materials science field.^78^ Moreover, in the Dong *et al.* (2022) research study, highly substituted poly(fluoropyrimidine)s were polymerized using diisocyanates, *N*,*N*′-diethyl barbituric acid, and dialdehyde without any catalyst. The researchers thoroughly investigated various experimental conditions, including solvents, temperature, and time. Through systematic optimization of these conditions, they succeeded in obtaining polymers with molecular weights as high as 16 400 g mol^−1^ and achieved excellent yields of up to 84%. The researchers also examined the thermal properties of the polymers and found that the decomposition temperature (*T*_d_, 5%) is 277 °C. Additionally, the structure of polyfluoropyrimidines may have been used in biopharmaceuticals.^79^ In order to synthesize a variety of polythioamides with well-defined structures and high molecular weight, Tang *et al.* explored this catalyst-free, 100% atom-productive procedure on a mixture of aromatic diynes, sulfur, and aliphatic diamines.^80^ By applying this catalyst-free method to a mixture of activated diyne, electrophilic styrene, and isocyanide for the preparation of highly substituted poly(cyclopentadiene), under mild conditions. High-molecular-weight polymers that are soluble and thermally stable can be produced in large quantities. A polymer with distinct AIE properties can be formed by adding a tetraphenylethene or triphenylamine moiety to its backbone. Interestingly, AIE polymers can also be produced *in situ* with monomers other than AIE. A photoresist can also serve as a visualizing agent to stain live cells' lipid droplets specifically.^81^ The metal-free polycycloaddition technique produces high molecular weights (*M*_w_ up to 166 000, *M*_w_/*M*_n_ = 3.20) soluble polytriazoles (PTAs) or neat conditions without protection from oxygen and moisture using an efficient perfluorophenylazide–alkyne polycycloaddition. The solvent used significantly impacts the regularity of the generated PTAs. Polymerizations in aromatic solvents like toluene and benzene yield more 1,4-regioisomers due to the arene–perfluoroarene interaction.^82^ Metal-free 1,3-dipolar polycycloadditions of 4,4′-isopropylidenediphenyl diphenylpropiolate and tetraphenylethene-containing diazides in dimethylformamide generate two soluble poly(phenyltriazolylcarboxylate)s (PPTCs) with huge molecular weights. These polymers, which are soluble in organic solvents and have a thermal stability of 5% at temperatures over 375 °C, can be used as fluorescent chemosensors and are highly effective for high-sensitivity explosive detection due to their luminous properties when aggregated.^83^ Furthermore, the Tang group developed a simple and effective method for thiol-yne click polymerization without external catalysts or heat. They mixed aromatic diynes 15a–e with dithiols 16 and 17 to create functional poly(vinylene sulfide)s (PVSs) with high molecular weight. PVSs with high regioregularity were only produced during polymerization. The molecular weight decreased dramatically when c-terpinene was added, indicating free-radical polymerization. PVSs are highly refractive in the 400–1600 nm wavelength region.^84^ These methods are utilized to generate heterocyclic heteroatom polymers.

### 2.3 Multicomponent cyclopolymerization (MCCP) based acetylenic synthesis of fused heterocyclic polymers

**Scheme 5:**
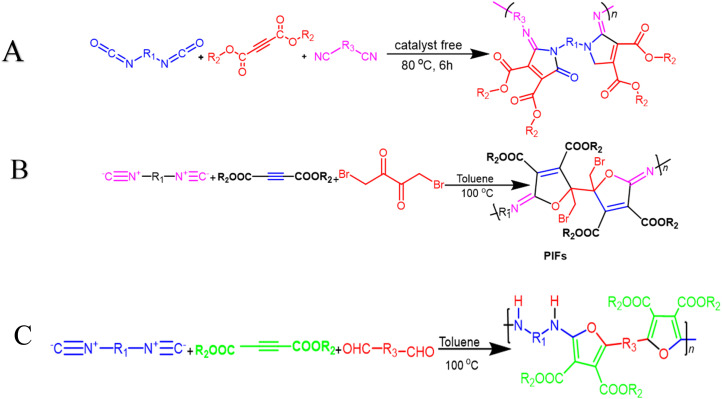
(A) Multicomponent cyclopolymerization of diisocyanatos, activated alkynes and diisocyanide (B) multicomponent cyclopolymerizations of diisocyanides, activated alkynes, and 1,4-dibromo 2,3-butanedione towards poly(iminofuran)s. (C) Synthetic route to poly(amine-furan-arylene)s.

Multicomponent cyclopolymerization (MCCP) offers advantages over other polymerization methods, including *in situ* fabrication of new ring units and one-pot heterocyclic polymer synthesis. It is widely used in coatings, photoelectric materials, controlled drug release, and gene transport processes, making it an effective and efficient method. In 2021, Wang *et al.* reported one-pot catalyst-free synthesis producing a series of soluble PMDs as poly(maleimide)s with excellent *M*_w_ values and high yields *via* activated alkynes, diisocyanides, and diisocyanates at 80 °C after 6 h (Scheme 5A). All these polymers have outstanding capabilities related to film-forming, good thermal stability with *T*_d_ up to 312 °C, and high refractive indices (RI) with values that range from 1.614 to 1.710 at 632.8 nm in their high-superiority thin films.^85^ In their study, Zhu Guinan, *et al.* offered MCCP of diisocyanides, activated alkynes, and 1,4-dibromo-2,3-butanedione 6 with a catalyst-free process, taking place in the presence of toluene under air for 6 h at a temperature of 100 °C. High-molecular-weight poly(iminofuran)s (PIFs) with well-defined structures were produced *in situ* in good yields (up to 89.5%) and at *M*_w_ up to 19 600 g mol^−1^ (Scheme 5B). The resilience related to this MCP was demonstrated by investigating various monomers that were present under optimal circumstances. The normalized UV absorption spectra of PIFs were then measured in THF due to the conjugated architecture of C=C that was connected to C=N in their backbone chains. It shows peaks related to absorption wavelength that were caused by the carbonyl group's n–π* transition additionally, cluster-triggered emission characteristics can be observed with polymers under UV irradiation. Thin films are usually made from PIFs that degrade quickly. Furthermore, the produced polymers that contain carboxylate and bromomethyl side chain decorations can easily be post-functionalized to establish polymers with star branched and biomedical constructed carriers, among other multifunctional materials. PIFs containing iminofuran rings have a prospective use in bioimaging functioning and therapeutics. They are also considered ideal lithographic materials, as they are easily degraded into low-molecular-weight volatile compounds when they are exposed to UV light.^86^ Fu and Weiqiang *et al.* used a catalyst free of diisocyanide, dialkylacetylene dicarboxylates, and dialdehyde to efficiently perform a mechanistic polymerization in toluene, resulting in high molecular weight poly(amine-furan-arylene)s. Modified furan-containing polymers give high atom economy with increased thermal stability and properties related to film processing (Scheme 5C).^87^ In the same manner, photodegradable poly(furan-amine)s (PFAs) series were synthesized from diisocyanide, dialkylacetylene dicarboxylates, and aromatic dialdehyde at 100 °C for 6 hours without inert gas protection. These PFAs have high refractive indices in the visible light range (400 nm to 800 nm) and can easily degrade into volatile products or residual fragments under ultraviolet light. Furan rings significantly degrade PFAs under UV irradiation, reducing over 90% of the film thickness.^88^

### 2.4 Green monomers based acetylenic synthesis of fused heterocyclic polymers

**Scheme 6:**
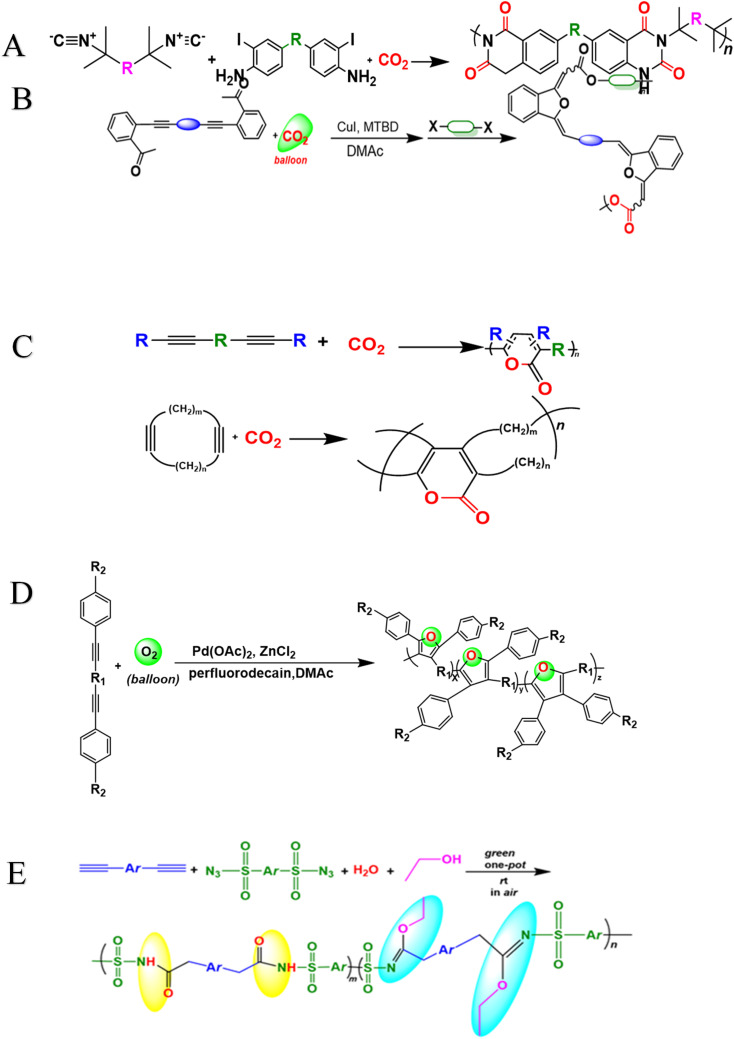
Synthesis of heterocyclic polymers derived from green monomers (CO_2_), (O_2_), (H_2_O) and triple-bond monomers.

As a natural, abundant, nontoxic, and environmentally friendly reagent, green monomers serve as a great resource to allow polymers to be created that will have functional properties.^89^ Monomers with triple bonds consist of alkynes, cyanides, and isocyanides, *etc.* The chemical properties of triple-bond monomers differ greatly from those of vinyl monomers.^90^ There have been numerous organic reactions using CO_2_ and triple-bond compounds; despite this, a few polymerizations have been investigated.^91^ The isocyanide monomer is a significant triple-bond monomer that can be utilized to create innovative CO_2_-based polymers. For example, Liu *et al.* (2021) developed a new multicomponent polymerization of CO_2_, diisocyanides, and bis(2-iodoaniline)s to synthesize new heterocyclic polymers (Scheme 6A). The polymers also had solubility and were thermally stable, and they self-assembled into spheres having a size range of around 200–1000 nm due to the interactions of hydrogen bonding related to amide groups present in the main chains. The polymers also had AIE features, which made them fluorescent probes that could be used to sensitively detect Au(iii) ions. The MCP was performed under 1 atm level of CO_2_ in a *N*,*N*-dimethylacetamide (DMAc) solution present at 80 °C for 18 h using PdCl_2_ and PPh_3_ as the catalyst precursors.^92^ A lower reactivity was observed for internal alkyne monomers when compared to terminal alkyne monomers. Recently, Song *et al.*^93^ succeeded in polymerizing CO_2_ and internal alkyne monomers using a CuI/7-methyl-1,5,7-triazabicyclo[4.4.0]dec-5-ene (MTBD) catalytic system (Scheme 6B). Carboxylation, cyclization, and esterification are considered three sequential processes that are mostly combined in the procedure. The MCTP reactions can easily be carried out in DMAc at atmospheric pressure, and the resultant poly(β-alkoxyacrylate)s usually exhibit increased temperatures related to the decomposition process and good thermal stability. Up to 96% of them were obtained with high yields. Such products also demonstrate the effect related to polymerization-induced emission (PIE) and the ability of non-luminescent monomers to become poly(alkoxyacrylate)s that are active in AIE.^93^ In addition, the polymerization of CO_2_ and internal alkyne monomers toward poly(2-pyrones) was demonstrated *via* Tsuda *et al.* In a mixture of THF and MeCN, nickel(0) complexes catalyzed the polymerization. The R9 group in the diynes is required to be aromatic or aliphatic, containing more than 5 carbon atoms, to avoid the intramolecular cyclization process. Under similar conditions, CO_2_ reacts with the cyclodiyne monomer to form ladder poly(2-pyrone) (Scheme 6C). The resulting poly(2-pyrone) has outstanding heat resistance, and under nitrogen gas, its decomposition temperature exceeds 420 °C.^94^ It is vital to utilize O_2_ efficiently as a monomer in the production of functional products. Qin *et al.* synthesized soluble poly(tetrasubstituted furan)s in *N*,*N*-dimethylacetamide and perfluorodecalin using Pd(OAc)_2_/ZnCl_2_. High yields of weight-average molecular weights (*M*_w_) were obtained (Scheme 6D), with two-photon absorption cross-sections up to 1570 g. These polymers have applications in optoelectronics and biology.^95^ Tang *et al.* (2017) described their work using MCP to synthesize polymers from a green monomer, diyne, disulfonyl azide, water, and ethanol. They found that this reaction could be catalyzed by using copper iodide (CuI) and triethylamine (Et_3_N) at room temperature. The resulting polymers had high molecular weights (up to 83 900 g mol^−1^) and good yields (up to 96%). The first attempt to polymerize diyne, disulfonyl azide, and water resulted in insoluble products. This was likely due to the extensive hydrogen bond formation that was present between the polymer chains, which caused them to crosslink. Adding alcohol to the mixture of reactions helped improve the polymer solubility by decreasing the hydrogen bonds. This was because the alcohol molecules could compete with the polymer chains for the hydrogen bonds, which weakened the interactions between the chains. Additionally, the flexible groups related to ethoxyl in the alcohol molecules also helped to increase the polymer solubility (Scheme 6E).^96^

### 2.5 Multicomponent tandem polymerizations (MCTP) based acetylenic synthesis of fused heterocyclic polymers

**Scheme 7:**
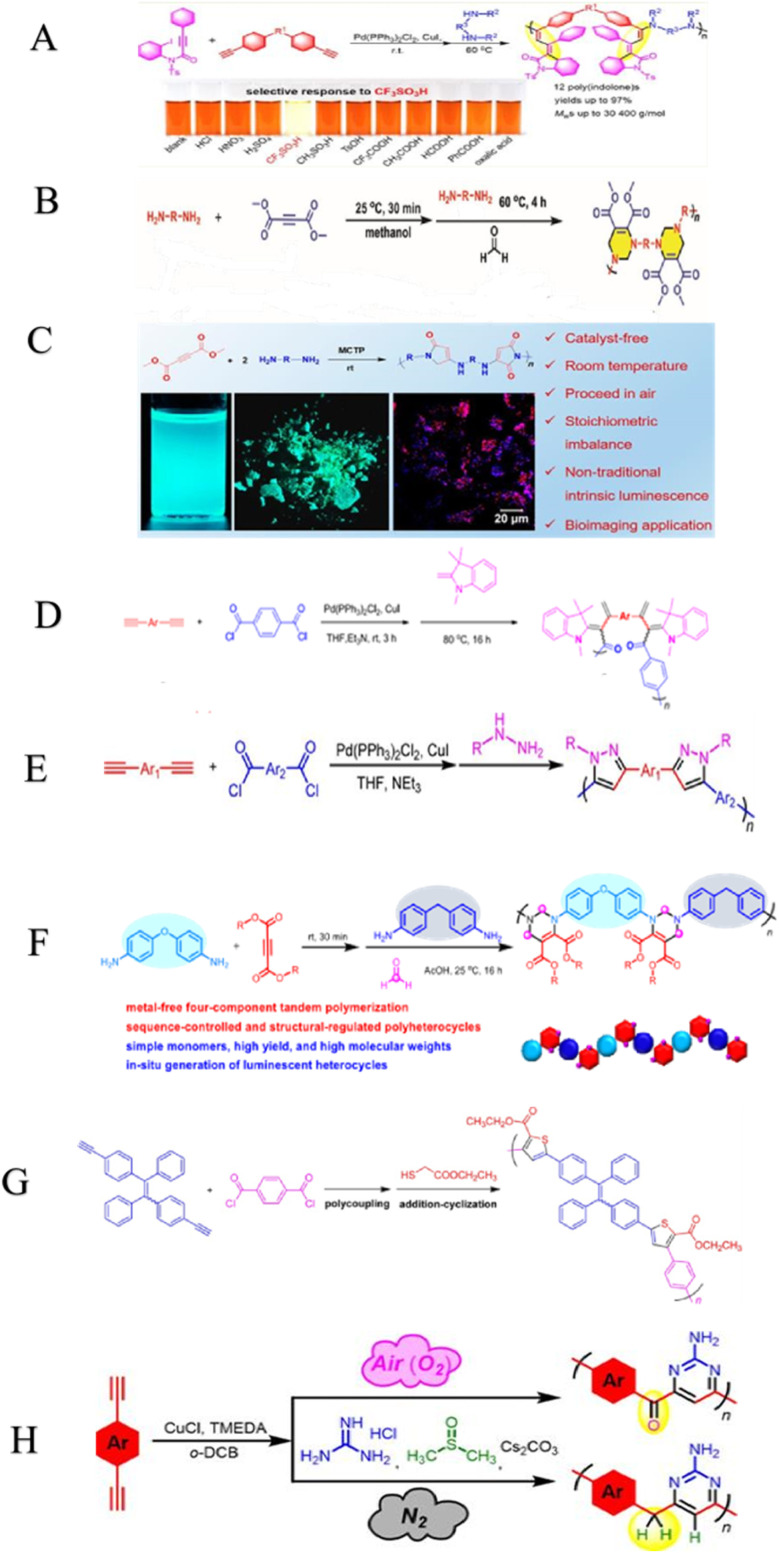
(A) MCTP of *N*-(2-iodophenyl)-3-phenyl-*N*-tosylpropiolamide, terminal diynes and diamines for the construction of poly(indolone)s. Acid-responsive to CF_3_SO_3_H. This figure has been adapted/reproduced from ref. 99 with permission from American Chemical Society, copyright 2019. (B) MCTP of aliphatic diamines, activated alkyne and formaldehyde. This figure has been adapted/reproduced from ref. 100 with permission from John Wiley and Sons, copyright 2023. (C) An investigation of the non-traditional intrinsic luminescent poly(aminomaleimide)s (PAMs), fluorescent images of PAMs in solution state and aggregate state, was linked with the merged confocal colocalization images of HeLa cells stained with PAMs. This figure has been adapted/reproduced from ref. 101 with permission from American Chemical Society, copyright 2020. (D) MCTP of aromatic alkynes, carbonyl chloride, and Fischer's base generates poly(diene merocyanine)s. This figure has been adapted/reproduced from ref. 102 with permission from John Wiley and Sons, copyright 2019. (E) Synthetic routes for polypyrazole. This figure has been adapted/reproduced from ref. 103 with permission from American Chemical Society, copyright 2016. (F) MCTP of alkynes, amines, and formaldehyde towards sequence-controlled polymers. This figure has been adapted/reproduced from ref. 104 with permission from American Chemical Society, copyright 2017. (G) Synthetic route toward poly(arylene thiophenylene) by Tandem polymerization. This figure has been adapted/reproduced from ref. 105 with permission from American Chemical Society, copyright 2014. (H) MCTP synthesis of structure-controlled pyrimidine derivatives and poly(pyrimidine)s. This figure has been adapted/reproduced from ref. 106 with permission from American Chemical Society, copyright 2018.

In MCPs, monomers and catalysts are combined in a single reaction system, resulting in a one step process. It is important to prevent interference between the components to minimize unwanted side-reactions. The development of efficient MCP systems is challenging due to the limited range of monomers and reaction options available.^97^ To overcome this limitation and enhance the versatility of MCPs, researchers have introduced multicomponent tandem reactions to improve the versatility of MCPs. These reactions offer high efficiency rates, accept multiple functional groups, and are atom-efficient. Unlike MCPs, MCTPs combine multiple steps in a predetermined order, producing sequential reactions without the isolation of reactive intermediates.^98^ For example, to synthesize 12 poly(indolone)s with a distinct architecture and high molecular weights (*M*_w_ up to 30.4 × 10^3^ g mol^−1^) in high yields, the MCTPs of *N*-(2-iodophenyl)-3-phenyl-*N*-tosylpropiolamide, aromatic terminal alkynes, and diamines were investigated. These polymers exhibit a distinct fluorescence “turn-on” response when exposed to acid (Scheme 7A).^99^ Recently, various studies have been made on heterocyclic polymers tandem polymerizations, such as poly(tetrahydropyrimidine)s, (PTHPs) that do not require a catalyst. In order to create PTHPs, Wei *et al.* (2023) examined the catalyst free MCTP creation by using aliphatic diamines, activated alkynes, and formaldehyde. The resultant PTHPs showed the well-defined structures, high molecular weights (up to 57 700 g mol^−1^), good yields (up to 84%), and satisfactory thermal stability from the MCTP, it was then carried out at 60 °C in the presence of methanol in air (Scheme 7B). Through the MCTP was then distinguished by the use of its available monomers, high efficiency rates, affordability, absence of catalyst and moderate operating conditions. This research has offered a quick and environmentally friendly method to create PTHPs with adjustable anchoring features this study highlights the advantages of the MCTP, including its catalyst-free nature, use of commercially available monomers, and ability to synthesize PTHPs with varying molecular weights and thermal stability.^100^ It is definitely possible to control polymer sequence structures by adjusting the feeding sequence depending on the reaction preference between the thiol/amino and vinyl/alkynyl groups. By controlling the sequence of alkyne and amine, functional polymers can be prepared easily and efficiently. The amino-yne click polymerization is also compatible with other reactions, which makes it suitable for multicomponent tandem polymerization coupled with ring-closing reactions for the synthesis of poly(aminomaleimide)s (PAMs) with non-traditional intrinsic luminescence characteristics (Scheme 7C). As fluorescent probes for bioimaging, the PAMs exhibited low cytotoxicity.^101^ Polypyrazoles containing heterocycles have been synthesized through a single-pot, one-step process utilizing the highly efficient MCTP of alkyne, carbonyl chloride, and hydrazines/aromatic diynes through a combination of Sonophashira coupling and Michael addition, resulting in a cyclocondensation reaction. High molecular weight (*M*_w_) up to 19 400 g mol^−1^ in high yields (Scheme 7D). Excellent solubility, processability, thermal stability, light transparency, refractive, and luminescence behavior are all characteristics of these polymers.^102^ Additionally, conjugated poly(diene-merocyanine)s with sufficient molecular weights (*M*_w_ up to 10.9 × 10^3^ g mol^−1^) and higher yields (up to 81%) were generated using one-pot MCTPs of alkynes, carbonyl chloride, and Fischer's base (Scheme 7E). Due to the diene merocyanine moiety being susceptible to AIEgen, it inherits polymers with high thermal stability and solubility, along with AIE activity.^103^ Wei *et al.* (2017) highlighted a new method for sequence-controlled luminescent polyheterocycles synthesis. The method is based on activated internal alkynes, aromatic diamines, and formaldehyde activation. The reaction of MCTP usually proceeds through various steps, incorporating the alkyne-amine click reaction, the imine-formaldehyde condensation reaction, and the cyclization reaction. The resulted polymers have increased molecular weights (up to 69 800 g mol^−1^) and high yields (up to 99%) (Scheme 7F). They demonstrate that the MCTP reaction can be used to synthesize a variety of different polyheterocycles, including tetrahydropyrimidines and dihydropyrroles. The polymers have inherent luminescence, which is due to the presence of the imine groups in the backbone. They also showed that monomers sequences in the polymer can be controlled by the sequence in which the monomers are added to the reaction mixture.^104^ Tang *et al.* investigated MCTPs for the fabrication of conjugated polymers (CPs). They discovered it's possible to produce functional CP materials from MCTPs by using the three-component reaction of alkyne, aryl chloride, and ethyl 2-mercaptoacetate catalyzed by Pd(PPh_3_)_2_Cl_2_ and CuI (Scheme 7G). Under mild room temperature conditions, it produces poly(arylene thiophenylene) with high yields (up to 97%) and molecular weights (*M*_w_ up to 156 000 g mol^−1^). The polymer was utilized as a delicate and specific fluorescent chemosensor for Ru^3+^, with quenching constants up to 8.8 × 10^5^ L mol^−1^, demonstrating the effectiveness of MCTPs in combining different processes under agreeable conditions into a single pathway related to polymerization, resulting in a polymer with various topologies.^105^ Hu and Tang *et al.* also reported the four-step MCTPs of diyne, guanidine hydrochloride, DMSO, and O_2_ that result in conjugated poly(pyrimidine)s (Scheme 7H). It is characterized by its well-structured and adjustable components. By combining Glaser coupling-nucleophilic addition–heterocyclization–oxidation reactions. These studies have shown the MCTPs' promising potential, which shows the ability to combine various reactions under favorable conditions into a single polymerization pathway to create polymers with a range of structures. The exploitation of such polymer architectures has significantly increased.^106^Table 1 summarizes heterocyclic polymers constructed by acetylenic multicomponent polymerizations and their applications.

**Table 1:** Heterocyclic polymers constructed by acetylenic multicomponent polymerizations and their applications

Name/structure	Catalyst ligand	Solvent	Base	Polymerization conditions			Applications/properties	Ref.
				Temperature	Yield	*M*_wt_		
Polytriazoles (1,4,5-PTAs)	Cu(MeCN)_4_BF_4_	DMF	*N*,*N*-Diisopropylethylamine (DIPEA)	Room temperature	95%	280 100 g mol^−1^	Laboratory explores macromolecular ligand/metal combinations	59
Azetidine-derivative heterocycles	CuI	Triethylamine (Et_3_N)	2,6-Lutidine	Room temperature	68%	13 600 g mol^−1^	Ion-responsive fluorescence	66
							Acid & base durable selective chemosensor for Pd^2+^ and Cr_2_O_7_^2−^	
Coumarin containing 1,4-polytriazoles	Copper(i) acetate	DMF	Cs_2_CO_3_	Room temperature	83–93%	20 080– 46 340 g/mol	Selective and efficient dye adsorbent	63
Poly(β-alkoxyacrylate)s	CuI	*N*,*N*-Dimethylacetamide	7-Methyl-1,5,7-triazabicyclo[4.4.0]dec-5-ene (MTBD)		96%	15 400 g mol^−1^	Undergo the conversion of CO_2_ into luminescent polymers shows aggregation-enhanced emission (AEE) property	93
Poly(phosphorus amidine)s	CuI	Tetrahydrofuran (THF)	—	Room temperature	92%	85 600 g mol^−1^	High refractivity with small chromatic dispersions; selective chemosensor for Pd^2+^ ion	50
Poly(imidazole)s	CuTC, Rh_2_(Oct)_4_	Chloroform	—	75 °C	83–98%	375.8 kDa	Optical properties and organic electronic materials	65
Functional oxazoline-containing polymers	CuI/PPh_3_/DIEA	Dichloromethane	—	Room temperature	97%	11 200 g mol^−1^	Fluorescent chemosensors for Fe^3+^ ion detection	58
Poly(diene merocyanine)s	Pd(PPh_3_)_2_Cl_2_, CuI	THF, Et_3_N	Fischer's base	80 °C	81%	10 900 g mol^−1^	Aggregation-enhanced emission (AEE) features	103
Poly(indolone)s	Pd(PPh_3_)_2_Cl_2_, CuI	THF	i-Pr_2_NEt	Room temperature	97%	30 400 g mol^−1^	Selective response to CF_3_SO_3_H	99
Poly(arylene thiophenylene)	Pd(PPh_3_)_2_Cl_2_/CuI	THF, Et_3_N	—	Room temperature	97%	156 000 g mol^−1^	Fluorescent chemosensor for Ru^3+^	105
Poly(*N*-sulfonylimidate)s	Cu(i), TEA	THF	—	Room temperature	96%	12 000 g mol^−1^	Responsive to various tumor microenvironment (pH, reducing reagent, ROS)	64
Poly(pyrimidine)s	CuCl, *N*,*N*,*N*′,*N*′-tetramethylethylenediamine (TMEDA)	*o*-DCB or DMSO	Cs_2_CO_3_	Room temperature	87%	25 300 g mol^−1^	Hydrophobicity; high thermal stability (*T*_d_ up to 529 °C)	106
Poly(amine-furan-arylene)s	—	Toluene	—	100 °C	91.2%	76 400 g mol^−1^	Black material	87

### 2.6 Multicomponent A^3^ coupling reactions based acetylenic synthesis of fused heterocyclic polymers

In the past few years, transition metal-catalyzed three-component reactions of aldehydes, alkynes, and amines, also known as A^3^ coupling, have become a powerful tool in organic synthesis. These reactions produce valuable N-containing heteroatom polymeric propargylamine products using an atom-economic approach. A great number of transition metal catalysts, such as Cu, Ag, and Au, have been used for the A^3^ coupling process.^107^ In order to construct fused-conjugated polymers with imidazo[2,1-*b*]thiazole units by using MCP related to heterocyclic diazoles, dialdehydes, terminal alkynes, or alkynecarboxylic acids, Cai Zhang and coworkers established a straightforward and efficient method. They improved various polymerization parameters, for instance, solvent, concentration of monomer, temperature, and catalyst quantity. It was then discovered that 0.05 M monomer present in the DMSO solvent with 0.2 equivalents of CuI catalyst, agitated at 80 °C for around 72 hours, the best combination used for the reaction. The polymerization process was executed smoothly, producing polymers were furnished with average yields and molecular weights of 5.7 × 10^4^ g mol^−1^. The resulting polymers, bearing the unique heterocycle section, displayed good solubility, significant optical activity, and low energy bands, indicating their prospective use in the photoelectric area.^108^ Further, Yan *et al.* developed a copper(ii)-bipyridine complex immobilized on amphiphilic polystyrene–poly(ethylene glycol) resin in 2019 as a catalyst for the three-component coupling of aldehydes, ketones, amines, and alkynes to produce propargylamines. Propargylamine was produced at a high yield of 96% under solvent-free conditions. Ketones can also be used in place of aldehydes, and the process was tested with various substrates.^109^ Bukowska *et al.* (2020) developed a method for synthesizing catalytic systems using a polymer gel with epoxy functionalities. The gel-type microscopic polymer beads were decorated with polyamidoamine-type dendrimers and heterocyclic aldehydes. The A^3^ coupling reaction of benzaldehyde, morpholine, and phenylacetylene was conducted using these polymeric supports. The polymeric catalysts with 2-pyridinecarboxaldehyde moieties effectively catalyzed these derivatives, and multiple recycling's of the material did not significantly impact its performance.^110^ Zarei *et al.* synthesized propargylamines on biochar@Cu–Ni nanocatalyst by A^3^ coupling reactions between aldehydes, amines, and terminal alkynes employing pomegranate shells, biochar was prepared at 500 °C and coated with Cu–Ni nanoparticles. Using nanocatalysts and toluene as solvents, the highest efficiency was achieved at 80 °C. The process of separating and recovering the nano-catalytic components was efficient, cost-effective, and straightforward. The novel feature of the catalyst is its Cu–Ni bimetallic structure, which provides a strong bond to the substrate. Propargylamine synthesis was conducted using an inexpensive, cost-effective catalyst.^111^ Furthermore, a new series of polymer-supported Ni(ii) complex catalysts with different ligand chain lengths for one-pot multicomponent A^3^ coupling processes is presented by Shi *et al.* These catalysts were prepared by a simple two-step process using commercially available polyacrylonitrile fibers. Longer-chain ligands in fiber-supported Ni(ii) complex catalysts showed higher efficiency than short chains, and their mediated reactions were flexible and smooth. The new fiber catalyst was scalable to the gram scale in a rotary core reactor and exhibited high strength, excellent flexibility, high stability, and recyclability.^112^ The study presents an efficient and sustainable method for synthesizing various new polymeric propargylamines in high-to-excellent yields. The method involves a three-component coupling of aldehydes or ketones with amines and alkynes, known as A^3^ or KA^2^ coupling, through employing a variety of monomers. This strategy can be applied to polymer synthesis, resulting in polymers with diverse properties and applications. The method uses a copper catalyst and undergoes a thermal degradation study to ensure high yields.^113^

### 2.7 Multicomponent passerini reactions for the synthesis of fused heterocyclic polymers

The Passerini reaction is a popular method for synthesizing monomers and functional polymers due to its mild reaction conditions and compatibility with various functional groups. It combines aldehydes, isocyanides, and carboxylic acids, offering a practical approach for functionalizing surfaces with numerous carboxylic acid groups. Commercially available isocyanides can introduce additional functional groups, making MCR an important part of the polymer synthesis portfolio. This process includes tandem and sequential reactions, producing individual products and becoming important components of synthetic combinations.^114^ Li *et al.* utilized the passerini reaction to create multiple linear functional poly(esteramide)s, a versatile class of polymers with a wide range of properties. They synthesized poly(esteramide)s with various functional groups, including hydroxyl, carboxyl, and amino groups, which can modify properties like solubility, biocompatibility, and conductivity.^115^ The authors successfully oxidized thioanisole and benzylamine using cross-linked polymers with Ru^3+^ complexes as efficient and recyclable heterogeneous photocatalysts through the Passerini reaction. MCRs are used to modify polymer characteristics due to high atom usage and structural designability.^116^ Wang *et al.* modified graphene oxide (GO) surfaces with polymers using the Passerini reaction in 2020 to enhance compatibility with 3D printing resins. Commercial GO was mixed with polymers with aldehyde-end functional groups in isocyanide. Various polymers were attached using grafting methods, with grafting density decreasing with longer chains. After blending with resin, mechanical stability improved by 0.1 wt% compared to unmodified GO.^117^ Lin *et al.* (2015) synthesized reduction-sensitive amphiphilic copolymers using a multi-component Passerini reaction between isocyanides, aldehydes, and carboxylic acids into the ester and amide linkages. These polymers self-assemble into micelles in an aqueous solution loaded with curcumin, a powerful anticancer agent. Transmission electron microscopy and dynamic light scattering confirmed the existence of nanoscale polymeric micelles. The polymer is reduction-sensitive, and cells primed with glutathione release curcumin more effectively. Curcumin-loaded micelles display better cellular proliferation inhibition than free curcumin, demonstrating their biocompatibility. *In vitro* experiments showed the micelles were biocompatible.^118^ Deng *et al.* (2014) developed a multi-component highly branched (HP) synthesis methodology, demonstrating the copolymerization of hexanedioic acid, hexane-1,6-dial, 1,6-diisocyanohexane, and 10-undecenoic acid using a one-pot ABC-type Passerini reaction. The structures were easily controlled, ranging from linear to hyperbranched.^119^ One area of research that demonstrates efficiency for the incorporation of multicomponent polymerizations involves photochemical processes. The modular nature of MCRs permits the introduction of photoresponsive moieties that may be added to provide a wide range of response or modification options. For instance, poly(ester-amide)s containing photolabile links in the backbone were produced by the Passerini addition polymerization of adipic acid, 1,6-diisocyanohexane, and photosensitive 2-nitrobenzaldehyde. The polymer backbone was completely broken down after 20 minutes of UV light on the polymer solution because the 2-nitrosobenzyl moiety was cleaved. The dicarboxylic acid in the Passerini-3CR was 3,3*-dithiodipropionic acid, which allowed for redox-initiated breakdown. It was also shown that orthogonal degradation might occur by UV light or redox initiation with dithiothreitol. Block copolymers were produced *via* the reaction of a monofunctional PEG acid with propargyl isocyanoacetamide and 2-nitrobenzaldehyde in a Passerini-3CR.^120^

### 2.8 Sequence-controlled multicomponent polymerization

**Scheme 8:**
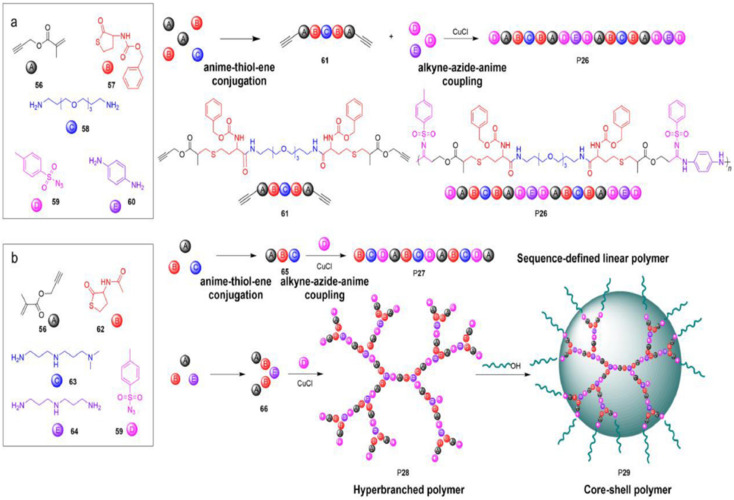
(a) Two consecutive multicomponent processes of amine-thiol–ene conjugating and alkyne–azide-amine couple in one pot (b) one-pot sequential reaction and multicomponent polymerization are techniques utilized to create sequence-controlled polymers. This figure has been adapted/reproduced from ref. 122 with permission from American Chemical Society, copyright 2015.

**Scheme 9:**
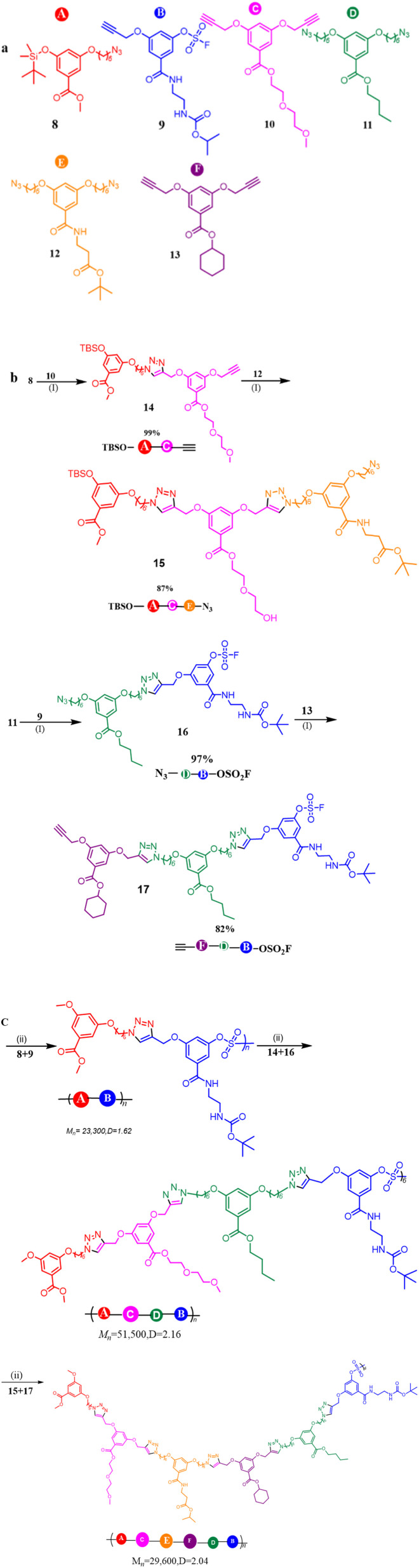
(a) Fabrication monomers to produce functional building blocks. (b) Synthesizing monomers based on predetermined sequence structures. (c) The approach of one-pot polymerization involves the production of polydispersed sequence-controlled polymers. This figure has been adapted/reproduced from ref. 124 with permission from John Wiley and Sons, copyright 2018.

Sequence-controlled polymer synthesis is a popular research field involving distinct repeating units and functions, such as proteins, DNA, and RNA, which are crucial for functionality and information storage. Special techniques like solid-phase peptide synthesis have been developed to mimic these polymers. It requires harsh reaction conditions and tedious experimental procedures to achieve solid-phase synthesis. MCPs, however, can combine three or more monomers in one step. MCP offers a novel method for synthesizing sequence-controlled polymers.^121^ For example, an innovative approach to generating synthetic sequence-controlled polymers was introduced by Zhang Ze *et al.* An alkyne, sulfonyl azide, and amine reaction and the conjugation of homocysteine thiolactone with primary amine were catalyzed by Cu in a single reaction pot. Propargyl methacrylate, an alkyne with an orthogonally electron-deficient carbon–carbon double bond, was utilized to integrate multi-component processes. In DMF at room temperature, homocysteine thiolactone was combined with diamine, propargyl methacrylate. The ABCBA segment was formed by adding diamine and *p*-toluenesulfonyl azide to a reaction solution enriched with 1,4-phenylenediamine and CuCl, with ABCBA, a difunctional monomer, serving as a key component. At 70 °C, the overnight reaction of three components through a Cu-catalyzed reaction led to the development of a periodic copolymer with a molecular weight of up to 163 200 g mol^−1^ (Scheme 8a).^122^ This has been successfully achieved using a method that combines direct multi-component polymerization advantages with the limitations of multicomponent reactions, resulting in a more diverse polymer backbone. After investigating, it has been determined that a novel molecule having *n*-acetylene-units and one secondary amine unit can easily be created in the initial amine-thiol–ene multicomponent reaction by substituting a primary diamine in the multicomponent cascade polymerization system with an amine having *n* (*n* ≥ 2) primary amine groups and a secondary amine group, such as *N*,*N*-dimethyldipropylenetriamine. Subsequently, all these resulting molecules undergo reactions with alkyne, secondary amine, and sulfonyl azide, which result in the formation of a polymer with an ABCD sequence structure. Moreover, the addition of various types of amines (Scheme 8b) enables the successful formation of polymers with hyperbranched and core–shell structures as well.^123^ Moreover, a recent click reaction based on the sulfur–fluoride exchange reaction (SuFEx) and fluor sulfate and sulfonyl fluoride as sulfonates or sulfonyl fluorides was employed for generating sequence-defined polymers. According to Cangjie Yang *et al.*,^124^ the orthogonal click chemistry of SuFEx and Cu-catalyzed Alkyne–azide cycloaddition (CuAAC) combined allows for the production of precision macromolecules with regulated alignment. The potential to produce sequence-defined polymers is further enhanced by the orthogonality of SuFEx and CuAAC click chemistry, which is insensitive to many other reactions with a wide variety of functionalities as illustrated in Scheme 9. Through their two methods, the authors illustrate the orthogonality of click chemistry. (a) Sequence-controlled polydisperse polymers with repeated sequence motifs are created using a stepwise growth polymerization technique. (b) Monodisperse oligomers with predetermined sequences are created by an iterative growth procedure without protective groups. Multiple click response strategies have been used in a variety of industries, including biomimicry, medicine delivery, and information encryption.^124^

Taking into consideration the findings of the aforementioned studies, Table 2 displays a summary of the different methods utilized for synthesizing heteroatom-functional polymers and developing alkyne-based compounds, with the advantages and disadvantages of each method provided.

**Table 2:** The advantages and disadvantages of different methods for synthesizing heteroatom-functional polymers and developing alkyne-based compounds

Methods	Advantages	Disadvantages	Ref.
Cu-catalyzed multicomponent polymerization	- Optimized consumption of starting materials enables diverse synthesis	- Residual copper catalyst removal is problematic and costly in copper-catalyzed reactions	49
	- To enhance the selectivity of the process, metallic catalysts		
Catalyst-free multicomponent polymerizations	- Green chemistry enables milder reactions for environmentally friendly synthesis	- Limited control, slow rates, narrow monomer range, lack of selectivity, optimization difficulties, limited scalability	70
	- Energy savings, safety, and controlled properties		
	- Cost-effective process & environmentally benign biodegradable polymer employing active metal free catalyst		
Multicomponent cyclopolymerization (MCCP)	- A highly effective enhanced structural diversity, efficient utilization of monomers	- Limited monomer availability and combinations, reaction optimization	88
	- Simplified reaction steps, and the potential for the synthesis of complex and functional polymeric materials	- Side reactions, reproducibility, and scalability challenges in polymer synthesis	
Green monomers-based acetylenic synthesis of fused heterocyclic polymers	- Natural, abundant, nontoxic, and environmentally friendly reagents, cheap, renewable, and sustainable features	- Optimize conditions, utilize green monomers, investigate mechanisms, study properties, scale up production	91
Multicomponent tandem polymerizations (MCTP)	- Step-economic MCTP transforms simple monomers into complex heterocycle-containing polymers, enabling diverse conjugated structures and advanced functionalities	- Low yield, high temperature, long reaction time, non-recyclable catalysts, and environmental concerns	100
Multicomponent A^3^ coupling reactions	- Mild reaction, diverse, and atom-economic incorporation	- Reactant selectivity and compatibility influence scalability and reproducibility	108
		- Extensive experimentation and optimization ensure consistent and efficient large-scale polymer synthesis	
Multicomponent passerini reactions	- Diverse structures, atom economy, mild conditions, simplified steps, and functional group compatibility	- Monomer availability, side reactions, optimization, structure control, scalability, and reproducibility for successful and controlled synthesis	114
Sequence-controlled multicomponent polymerization	- Precise sequence control, diverse monomer incorporation, orthogonal reactivity, modular approach, and customizable properties	- Synthetic complexity, limited monomer availability, reaction optimization, side reactions, reproducibility, scalability, cost, and efficiency	121

## 3. Conclusion

To sum up, we have provided an outline of the most recent advances in novel strategies using the fabrication of alkyne monomers regarding heterocyclic polymers with differing structures and functions. In addition, the use of catalyst-free alkynes metathesis polymerization transition-metal catalyst systems is also explored in the study. New metal-free acetylenic monomers offer methods for forming functionally fused or conjugated heterocyclic polymers. Multicomponent polymerization, a straightforward one-pot or sequential process, creates complex but well-defined structures, making it a popular research area. The development of MCPs involving green monomers presents both opportunities and challenges. Expanding green monomer types, improving polymerization reactions, exploring mechanisms, studying polymer properties, and requiring industry production scale-ups are necessary for the field's growth. Sequence-controlled polymer synthesis is a novel method for multicomponent alkyne polymerization, advancing biomimetic polymers and boosting optimism in the future of acetylene polymer chemistry, influenced by global polymer chemists' achievements. More research is needed to develop approaches based on alkyne polymer chemistry, including alkyne-controlled and living polymerizations, formed polymers with advanced optoelectronic properties, and engineered biopolymers. Alkyne polymerization research is a challenging but rewarding field that benefits various fields like materials science and biochemistry. MCP reactions, which use monomers like alkynes, isocyanides, amines, aldehydes, and ketones, offer several advantages over traditional polymerization methods. They are efficient, atom-economic, and environmentally friendly, making them valuable for developing new materials with diverse applications. However, more work is needed to establish these approaches. Notwithstanding the previously mentioned obstacles and restrictions, there are numerous valuable perspectives and potential future paths that may be seen. The subject of one-pot multicomponent polymerization for the synthesis of heterocyclic polymers shows great promise and offers interesting opportunities for research. Future developments can be driven by focusing on important areas such as the expansion of monomer combinations, precise adjustment of polymer characteristics, implementation of functionalization strategies, investigation of applications, and optimization and scale-up of processes. Further investigation and cooperation in these areas will reveal the complete capabilities of this field, allowing for the creation of innovative materials with customized characteristics for a diverse array of uses.

## Conflicts of interest

There are no conflicts to declare.

## Supplementary Material
